# Maintenance of certification for radiologists: an overview of European countries

**DOI:** 10.1186/s13244-020-00893-4

**Published:** 2020-07-17

**Authors:** Robert M. Kwee, Thomas C. Kwee

**Affiliations:** 1 Department of Radiology, Zuyderland Medical Center, Heerlen/Sittard/Geleen, The Netherlands; 2 Department of Radiology, Medical Imaging Center, University Medical Center Groningen, University of Groningen, Hanzeplein 1, P.O. Box 30.001, 9700 RB Groningen, The Netherlands

**Keywords:** Licensure, Education, Continuing, Certification, Radiology

## Abstract

**Background:**

It is currently unclear whether the continuing medical education (CME) requirements for radiologists to keep up their certification are equal across Europe, which would be desirable for uniform cross-border quality of radiology and because of the fundamental principle of free movement of workers in the European Union. This study aimed to determine the maintenance of certification requirements for radiologists in different European countries.

**Methods:**

National radiological societies of European countries and/or their delegates as listed on the European Society of Radiology website were contacted to inquire about the maintenance of licensure requirements for radiologists in their country. Data were analysed using descriptive statistics.

**Results:**

Forty-six European countries were contacted. Response rate was 80%. Twenty-two of 36 responding countries (59%) reported mandatory requirements to maintain a radiologist’s license to practise. The median license period was 5 years (range 1–7). The median required number of CME points per year was 40 (range 8–58, interquartile range 30). Eight countries reported additional requirements, including practising clinical radiology, attending quality meeting/clinical audit, and attending additional courses (such as radiation safety training and advanced medical training course). Fifteen of 37 responding countries (41%) did not report mandatory requirements.

**Conclusions:**

There is considerable heterogeneity across European countries regarding the maintenance of certification requirements for radiologists. More homogeneity is desired for uniform quality assurance and professional mobility of radiologists across Europe. The data from our overview may be used to establish a benchmark for national societies who issue maintenance of licensure requirements for radiologists.

## Key points

- Fifty-nine percent of European countries have mandatory requirements to maintain a radiologist’s license to practise.
- In these European countries, radiologists are required to obtain at least 8 up to 58 (median 40) CME credits per year.
- Standardisation of maintenance of certification requirements is desired for more uniform quality and interchangeability of radiologists across Europe.

## Introduction

Radiologists have to undergo intensive training and assessment in order to get accredited by pertinent governing bodies [1]. In Europe, radiology training takes on average 5 years (range 2–6 years), whereas subspecialty fellowship training is offered in just over half of European countries [1]. The ESR has defined the training requirements for trainees in radiology [2]. In 2011, the European Society of Radiology (ESR) created the European Diploma in Radiology (EDiR), which serves the standardisation and accreditation of radiologists across European borders [3, 4]. From the times of Hippocrates, medical doctors have taken oaths to keep their knowledge and skills up-to-date [5]. The importance of keeping up with knowledge and new developments is self-evident to maintain an adequate level of patient care. As progress in medicine becomes ever faster, the necessity to update ones knowledge is even greater [5]. This especially holds true for radiology, where new knowledge is being developed at an increasingly rapid rate owing to technological advances [6, 7]. The process of continuing medical education (CME) is a part of every physician’s professional growth, development, and lifelong learning [8, 9]. It aids the radiologist in keeping current with new techniques, procedures, and information [8]. To our knowledge, however, it is not clear yet whether the CME requirements for radiologists to keep up their certification are equal across Europe. Equal CME requirements would be desirable for uniform cross-border quality of radiology and because of the fundamental principle of free movement of workers in the European Union (EU) [10]. Therefore, the purpose of our study was to determine the maintenance of certification requirements for radiologists in different European countries.

## Methods

Ethics committee approval was not applicable for this study. This study was driven by personal interest and not an ESR initiative. The authors who analysed and interpreted the data (R.M.K. and T.C.K.) had no conflicts of interest with regard to this study.

### Data collection and analysis

National radiological societies of European countries and/or their delegates as listed on the ESR website [11] were contacted by email and asked the following single question: “What are the requirements for a radiologist to keep his/her license to practise in your country (e.g. required continuing medical education [CME] points per which time period, minimum number of working hours, etc.)?” Although some countries are geographically not located in Europe but in Asia, they are all ESR member societies and tend to join the ESR activities. These countries were also included under the umbrella of “Europe at large.” Emails were initially sent out mid-January 2020. In case of no initial response, repeated emails were sent up to two times within 1 month. In our analyses, 1 h of reportedly required educational activity was regarded to correspond to 1 CME credit [12]. Data were analysed using descriptive statistics.

## Results

**Fig. 1:**
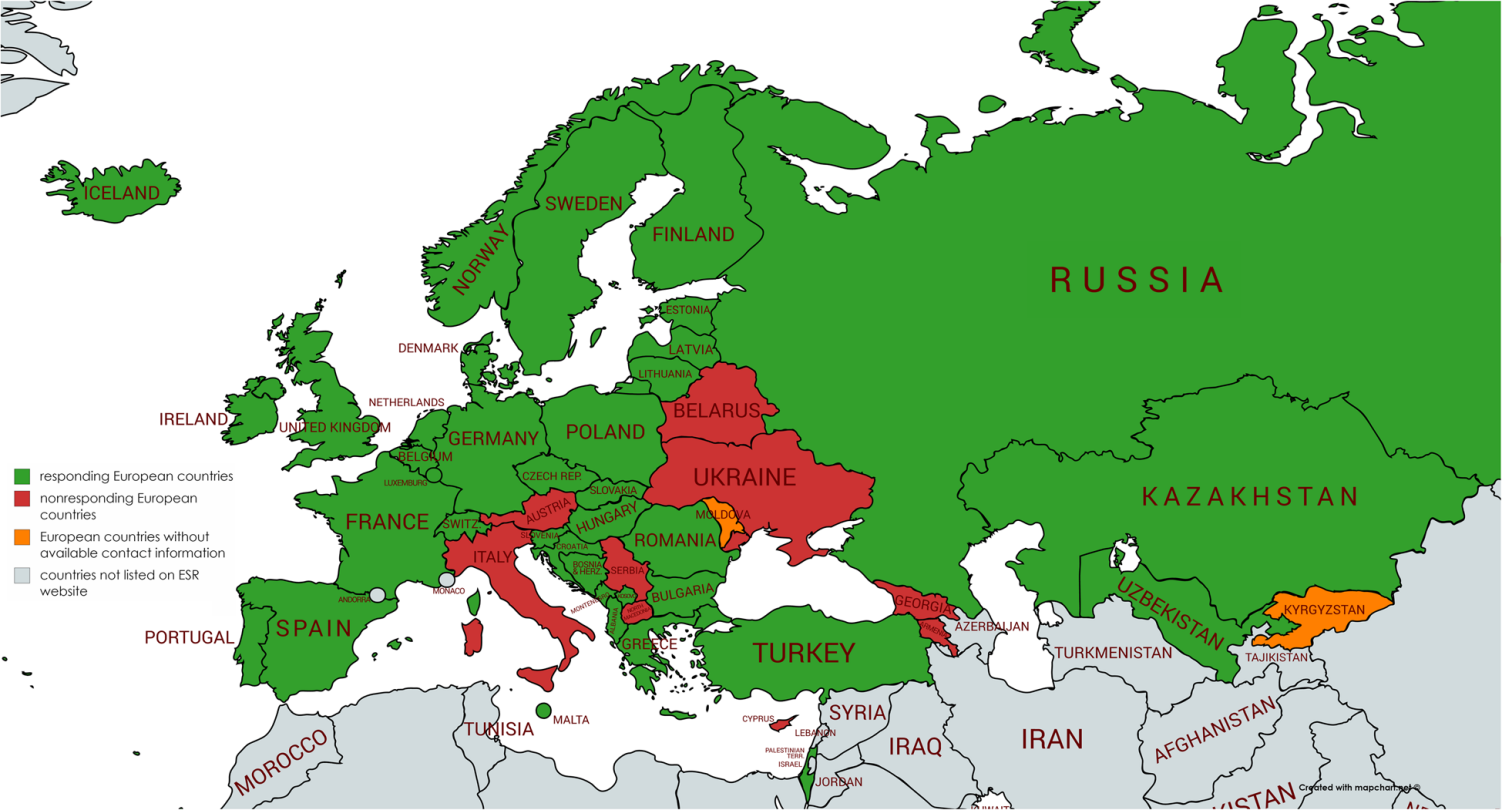
Overview of responding European countries, non-responding European countries, and European countries without available contact information

Forty-six European countries were contacted. Contact email addresses of Kyrgyzstan and Moldova were not available. Responses were received from 37/46 national radiological societies of European countries (80% response rate) (Fig. 1), which were included in the analyses. An overview of the responses is displayed in Table 1. Twenty-two of 37 responding countries (59%) reported mandatory requirements to maintain a radiologist’s license to practise. Accordingly, 15 of 37 responding countries (41%) did not report mandatory requirements. The median license period was 5 years (range 1–7). The median required number of CME points per year was 40 (range 8–57.6, interquartile range 30). Eight countries reported additional requirements, including practising clinical radiology, attending quality meeting/clinical audit, and attending additional courses (such as radiation safety training and advanced medical training course) (Table 1).

**Table 1:** CME and additional requirements for a radiologist to keep his/her license to practise per country

Country	Required number of CME credits	Additional requirements	License period	Average CME credits required per year
Albania	250	Practising clinical radiology for 40 h per week	5 years	50
Austria	Unknown (no response)	Unknown (no response)	Unknown (no response)	Unknown (no response)
Armenia	Unknown (no response)	Unknown (no response)	Unknown (no response)	Unknown (no response)
Belarus	Unknown (no response)	Unknown (no response)	Unknown (no response)	Unknown (no response)
Belgium	-6 (required for federal agency for nuclear control, mean 2 per year) -60 (other, of which at least 9 [mean 3 per year] in ethics and economy)	-Attending 2 local quality group meetings per year -Production performance of at least 1250 “prestaties”*	3 years	22
Bosnia and Herzegovina	90	None	5 years	18
Bulgaria	None	None	NA	NA
Croatia	120	None	6 years	20
Cyprus	Unknown (no response)	Unknown (no response)	Unknown (no response)	Unknown (no response)
Czech Republic	None	None	NA	NA
Denmark	None	None	NA	NA
Estonia	None	None	NA	NA
Finland	40 (radiation safety training)	None	5 years	8
France	None	None	NA	NA
Georgia	Unknown (no response)	Unknown (no response)	Unknown (no response)	Unknown (no response)
Germany	200	- Attending 1 radiation safety training per year - Attending 1 refresher course in radiation safety per 5 years	5 years	40
Greece	None	None	NA	NA
Hungary	250	Practising clinical radiology without any specific requirements	5 years	50
Iceland	None	None	NA	NA
Ireland	50 (minimum 20 external [maintenance of knowledgeand skills], minimum 20 internal [practice evaluation and development], minimum 5 personal learning, and a desired 2 in research and teaching)	Attending 1 clinical audit (equals 12 CME credits)	1 year	50
Israel	None	None	NA	NA
Italy	Unknown (no response)	Unknown (no response)	Unknown (no response)	Unknown (no response)
Kazakhstan	108 to 216 (depending on the specialties required: radiography, ultrasound, CT/MRI, and/or nuclear medicine)	None	5 years	21.6 to 43.2
Kosovo	100	None	5 years	20
Kyrgyzstan	Unknown (no contact information available)	Unknown (no contact information available)	Unknown (no contact information available)	Unknown (no contact information available)
Latvia	250 (at least 60% in radiology)	None	5 years	50
Lithuania	120	None	5 years	24
Luxembourg	None**	None	NA	NA
North Macedonia	Unknown (no response)	Unknown (no response)	Unknown (no response)	Unknown (no response)
Malta	None	None	NA	NA
Moldava	Unknown (no contact information available)	Unknown (no contact information available)	Unknown (no contact information available)	Unknown (no contact information available)
Montenegro	120 (at least 72 in radiology, the number of CME points accumulated in one calendar year may not be less than 10)***	None	7 years	17.1
The Netherlands	200 (with a maximum of 50 that can be counted in from continued education provided by other non-imaging medical specialties)	- Practising clinical radiology at least 16 h per week -Participation in individual performance evaluation -Attending 1 clinical audit	5 years	40
Norway	None	None	NA	NA
Poland	25	None	1 year	25
Portugal	None	None	NA	NA
Romania	200	None	5 years	40
Russia	250	None	5 years	50
Serbia	Unknown (no response)	Unknown (no response)	Unknown (no response)	Unknown (no response)
Slovakia	250****	None	5 years	50
Slovenia	75	Practising clinical radiology without any specific requirements	7 years	10.7
Spain	None	None	NA	NA
Sweden	None	None	NA	NA
Switzerland	150 (at least 50% in radiology)	None	3 years	50
Turkey	None	None	NA	NA
Ukraine	Unknown (no response)	Unknown (no response)	Unknown (no response)	Unknown (no response)
United Kingdom	250	None	5 years	50
Uzbekistan	288	Attending 1-month training course at the Institute of Advanced Medical Training (equals 144 CME credits)	5 years	57.6

*CME* continuing medical education, *NA* not applicable

*Not strictly compulsory, but gives financial benefits (e.g., higher reimbursement of radiologic examinations)

**The only exception concerns the national program for breast cancer detection where the radiologists have to read at least 1000 mammograms in this program and do 8 h of CME every year in this specific area

***If a radiologist earns more than 120 CME points during the license period, he/she may transfer 10 points to the next license period

****Radiologists can get 100 CME credits for a minimum of 4 years of continuous work in radiology, if less than 4 years, then 25 points for every year completed

## Discussion

Our overview shows that there is a wide variation with respect to requirements to maintain a radiologist’s license to practise: in 22 of 37 responding European countries, radiologists are required to obtain at least 8 up to 50 (median 40) CME credits per year, whereas in other European countries, radiologists currently keep their license for life without any mandatory CME requirements. Remarkably, such a considerable heterogeneity with regard to certification maintenance has also been reported for other medical specialties [13–16].

In 2006, Bresolin et al. [17] conducted a survey on maintenance of certification in radiology among 34 countries worldwide [17]. At that time, CME was requested for radiologists in 13 of 24 responding countries (54%) and requested under certain circumstances in another four countries (17%) [17]. Interestingly, one third of countries failure to meet the requested CME requirements would not lead to loss of licensure or certification [17]. It should be noted, though, that there is still no evidence of a causative link between improved health care outcomes and either mandatory or voluntary recertification systems [15]. It is difficult to compare the study by Bresolin et al. [17] to our current study, because Bresolin et al. [17] did not report how many CME credits were required per country and because they included responses of only 7 European countries (Austria, Belgium, Germany, Ireland, Romania, Spain, and the UK) [17]. Nevertheless, our study shows that Belgium, Germany, Ireland, and the UK currently have mandatory requirements (minimum number CME credits to be obtained and/or participation in quality improvement types of programs). This suggests a trend towards an increased number of European countries with official requirements to maintain a radiologist’s license to practise. Accordingly, some Scandinavian countries also responded that they are planning to formalise CME requirements.

Europe can be compared to the USA in terms of size: both consist of multiple countries (Europe) or states (USA). However, the requirements for maintenance of certification for radiologists in the United States of America (USA) are more uniform than among European countries. Although every state medical board has slightly different CME requirements [18], the umbrella organisation for radiologists in the USA, the American Board of Radiology (ABR), requires radiologists to attain 75 CME credits every 3 years to satisfy maintenance of certification. At least 25 of these 75 CME credits must be from self-assessment CME activities, which are primarily podium presentations with a post-session assessment instrument [19]. In addition, besides having a valid and unrestricted licensure in all states, radiologists should pass the ABR’s online longitudinal assessment and complete at least one practice quality improvement project or participatory quality improvement activity every 3 years [19]. All the provinces in Canada have equal CME requirements for radiologists [20], which is also the case in Oceania [21]. More uniformity in Europe would be desirable for uniform radiology quality and professional mobility of radiologists across European countries. However, the current systems of licensing and registration of medical doctors within the EU, which are controlled by national regulatory bodies, are diverse and complex [13–16]. The ESR could, similar to the EDiR initiative [3, 4], play a future leading role to achieve uniformity in maintenance of certification for radiologists across Europe.

Our study has some potential limitations. First, we did not receive a response from all countries. However, the response rate of 80% can be considered high [22, 23]. Moreover, even a 100% response rate would not change the main result. Second, our study presents a current overview of national regulations, which may change in the near future. Third, 8 European countries reported additional requirements besides CME, including practicing clinical radiology, attending quality meeting/clinical audit, and attending additional courses (such as radiation safety training and advanced medical training course), as listed in Table 1. However, it was not possible to provide comparable summary measures of these additional non-CME requirements because of their heterogeneous nature. Nevertheless, they should be taken into account when comparing maintenance of certification requirements among different countries.

In conclusion, our overview shows that there is considerable heterogeneity across European countries regarding the maintenance of certification requirements for radiologists. More homogeneity is desired for uniform quality assurance and professional mobility of radiologists across Europe. The data from our overview may be used to establish a benchmark for national societies who issue maintenance of licensure requirements for radiologists.

## Data Availability

Available on request.

## Abbreviations

CME: Continuing medical education
EDiR: European Diploma in Radiology
ESR: European Society of Radiology
EU: European Union
